# Assessment of the effects of exposure to extremely low-frequency magnetic fields on MDCK epithelial cell lines under a controlled environment

**DOI:** 10.1093/jrr/rrab001

**Published:** 2021-02-17

**Authors:** Gonzalo Domínguez, Eladio Cardiel, Elsa Sánchez, Pablo-Rogelio Hernández

**Affiliations:** Department of Electrical Engineering, Bioelectronics section, Center for Research and Advanced Studies of the National Polytechnic Institute, Mexico City, Mexico; Department of Electrical Engineering, Bioelectronics section, Center for Research and Advanced Studies of the National Polytechnic Institute, Mexico City, Mexico; Department of Physiology, Biophysics, and Neuroscience, Center for Research and Advanced Studies of the National Polytechnic Institute, Mexico City, Mexico; Department of Electrical Engineering, Bioelectronics section, Center for Research and Advanced Studies of the National Polytechnic Institute, Mexico City, Mexico

**Keywords:** magnetic field exposure, MDCK, controlled environment, TEEI, cell migration, immunofluorescence

## Abstract

To assess the effects of exposure to extremely low-frequency magnetic fields (ELF-MFs) on MDCK cell lines, experiments were performed in a chamber under controlled conditions (temperature, humidity and CO_2_). Therefore, the measured physicochemical and electrical changes in the cells are due solely to the magnetic field exposure and not to external factors. A developed sinusoidal magnetic field generator produced the ELF-MFs with a uniform magnetic field and adjustable intensity and frequency. Three experimental indicators were used: (i) transepithelial electrical impedance (TEEI); (ii) cell migration and proliferation; and (iii) expression of the proteins of the tight junctions, and changes in the area and shape of the cell nuclei. No significant effects on TEEI values were observed when 10 and 50 G 60 Hz magnetic fields were applied to confluent cell monolayers. There were no significant differences in migration and proliferation of the cell monolayer exposed to 60 Hz magnetic fields10 and 50 G , but a contact inhibition factor was observed. The expression of the CLDN-1 protein decreased by 90% compared with the control, while ZO-1 protein expression increased by 120%. No significant effects were observed in the area and shape of the cell nuclei. Experimentation in a controlled environment, under physiological conditions, ensures that the observed effects were strictly due to exposure to magnetic fields. Different exposure conditions are necessary to determine the impact on TEEI and cell migration–proliferation indicators.

## INTRODUCTION

Magnetic fields surround everything; therefore it is essential to study their effects on living organisms [1]. Several studies have investigated this; however, elucidating the effects at the cellular level remains challenging. Electromagnetic radiation interacts with biological matter [2], as demonstrated by electric power transmission lines in urban areas, and the increasing use of electric motors and household appliances. The population is increasingly exposed to low-frequency magnetic fields, causing a corresponding rise in potential health risks and mortality [3]. The International Commission for Protection against Non-Ionizing Radiation (ICNIRP) has established limits for exposure to electromagnetic fields (EMFs) in workplaces and residential homes. They recommend short periods of exposure to extremely low-frequency magnetic fields (ELF-MFs) to avoid detrimental health effects [4].

There are a number of epidemiological reports related to magnetic field exposure [5], as well as treatments that utilize this radiation for wound cell restoration [6], bone formation [7], pain relief for osteoarthritis [8] or for the care of some types of brain cancer and leukemia [9, 10].

A number of *in vitro* experiments have been conducted to study the effects of magnetic field exposure, at the cellular or tissue level, e.g. changes in Ca^2+^ flux, cell growth and proliferation, and in DNA, RNA and protein synthesis [11–13]. However, no definitive conclusions on these issues have been made. Therefore, in this study, an experimental protocol was designed using epithelia, *in vitro*, exposed to magnetic fields, under controlled conditions.

Canine Madin–Darby kidney (MDCK) cell cultures were used in this study: these model epithelium cells are commonly used to study the regulation of cell growth, metabolism and transport mechanisms [14–17].

The evaluation of the effects of magnetic radiation on cell lines was carried out using three indicators. First, the transepithelial electrical impedance (TEEI) due to the natural relationship among magnetic fields, ion dynamics and electrochemistry of cell membranes. Second, cell migration was determined, because tissue regeneration and cancer cell growth inhibition are altered by exposure to magnetic fields [18]. Migration and proliferation phenomena were observed using a scratch-wound assay [19–21], which gives information on the rate of wound regeneration, determined by the percentage of confluence [22]. Third, the expression of claudin and occludin proteins in tight junctions was analyzed because of their contribution to epithelial barrier function in MDCK cells [23]. Protein expression was determined by analyzing images obtained by immunofluorescence with confocal microscopy. In addition, it has been reported that the shape, size and quantity of cell nuclei are modified with a stimulus [24].

## MATERIALS AND METHODS

MDCK (NBL-2) (ATCC-CCL-34) cell lines were used as an experimental model formed in a cell monolayer. A mean of 25 000 cells per well was taken from the same strain and seeded (under normal growth conditions) in a sterile 6.5 mm Transwell®-COL collagen-coated 3.0 μm pore polytetrafluoroethylene (PTFE) membrane insert (Corning 3496, Mexico). The inserts were left in the culture medium, to be moistened 1 day prior to seeding the cells. The medium (150 μl) plus 4.02 μl of cells was placed in the well and 1500 μl of the medium outside. The cell culture medium was prepared with Dulbecco’s modified Eagle’s medium (DMEM-high glucose D5648, Sigma-Aldrich, Mexico), 10% fetal bovine serum (FBS; Gibco, Mexico), 10 000 IU ml^–1^ penicillin and 20 mg ml^–1^ penicillin–streptomycin (Sigma-Aldrich, Mexico). Cells were grown on three different substrates, according to the experimental protocol indicators.

Cell cultures were exposure and control pair-grouped. All preservation and handling processes were performed in a controlled environment, inside a chamber, where temperature, CO_2_ and humidity were regulated. The renewal of medium was also done in the chamber to avoid hypoxia and changes in light and temperature.

The magnetic field generator, custom-developed for this project [24], and the impedance meter were designed to be applied inside the chamber. The sinusoidal ELF-MF source was developed expressly for this project. It was implemented with a Helmholtz coil array, powered by a TDA2030 amplifier (STMicroelectronics, USA) and controlled by a digital system based on a DAQ card (NI-USB 6218; National Instruments, USA). The digital system also included a circuit for regulating the magnetic flux intensity changes and monitoring the temperature when the frequency changes. A uniform magnetic field and a maximum temperature increase of 0.4°C were observed after 12 h exposure to 60 Hz at 50 G in the working volume [24, 25].

The magnetic field generator was validated by measuring its main parameters in the working chamber, including the value of the magnetic field density, frequency, uniformity and the direction of the magnetic flux. A sterile environment was maintained within the chamber, with the temperature maintained at the recommended 36.5 ± 0.5°C in the working area, measured using a temperature sensor (LM35) during the 12 h of operation.

The experiment was designed to use 10 and 50 G, peak to peak over zero of magnetic field density and 60 Hz frequency, to identify significant electrical and physical changes on epithelia.

The experimental protocol considered three indicators: (i) TEEI, which includes transepithelial electrical resistance (TEER) and cellular capacitance (Ccl); (ii) cell migration and proliferation using the scratch-wound assay; and (iii) protein expression of the tight junctions, and the changes in cell density and physical characteristics of the cell nucleus of the epithelium, determined by immunofluorescence.

### TEEI

**Fig. 1.**
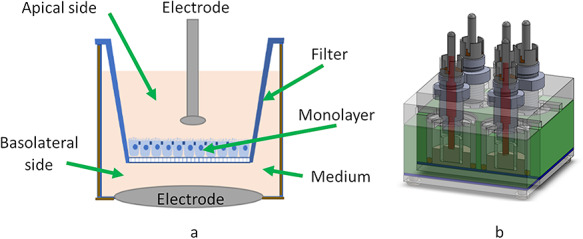
(a) Transepithelial electrical impedance (TEEI) measurement chamber based on the EndOhm arrangement; (b) multiwell measuring chamber, based on the arrangement proposed by Wegener.

To measure the TEEI, cells were grown in permeable filters, which allowed the monolayer to have contact with the medium on both apical and basolateral sides (Fig. 1a). To guarantee a sufficient number of samples for the measurement of electrical impedance of the monolayers, a multiwell measuring chamber was designed, following the arrangement proposed by Wegener [26]. This consisted of four independent wells, housing inserts with permeable filters to attach the cell cultures, a stainless-steel plate on the base as a reference electrode, and four recording electrodes on the upper part of the wells immersed in the culture medium on the apical side of the monolayer. PTFE also known as Teflon, was chosen for the construction of the four-well block because it is non-polluting and an electrical insulator. The wells were sealed with silicone O-rings. This arrangement is shown in Fig. 1b.

To determine the TEEI, sinusoidal magnetic fluxes at 60 Hz of 10 and 50 G were applied for 60 min every 4 h, over 24 h (six exposures). This exposure time was used to avoid cell temperature effects, due to the inherent heating of the generator. Twelve electrical impedance measurements were made before and after each exposure.

### Scratch-wound assay

For the scratch-wound assay, cell cultures were grown in Petri dishes until they reached confluence. Under these conditions, a wound was made and exposed to ELF-MFs. The test was carried out by making a wound in the confluent cell monolayer with a pipette tip to assess the phenomena of cell migration and proliferation. The evolution of the regeneration process was recorded by images taken every 120 min in a light microscope with a Moticam 2000 high-resolution camera and Motic image software [Motic (Xiamen) Electric Group Co. Ltd, China].

**Fig. 2.**
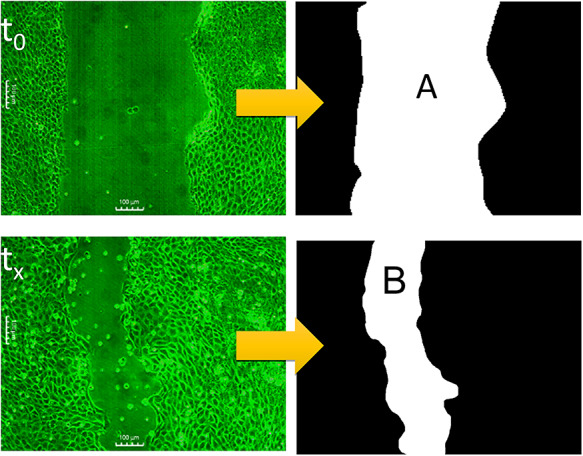
(a) Wounds images taken by the confocal microscope; (b) binarized images (from MATLAB) to calculate the percentage area covered by cells.

There were six magnetic field exposures at 60 Hz, with an intensity of 50 G, applied every 4 h with a duration of 60 min, over 24 h. Images were taken every 2 h. To improve the quantification of the confluence percentage, a program was developed in MATLAB (MathWorks, Natick, MA, USA) to binarize the cell images in order to determine the area of the wound from the number of white pixels. Figure 2 shows an example of the binarized images, corresponding to the cell confluence process of the scratch-wound assay**.**

**Fig. 3.**
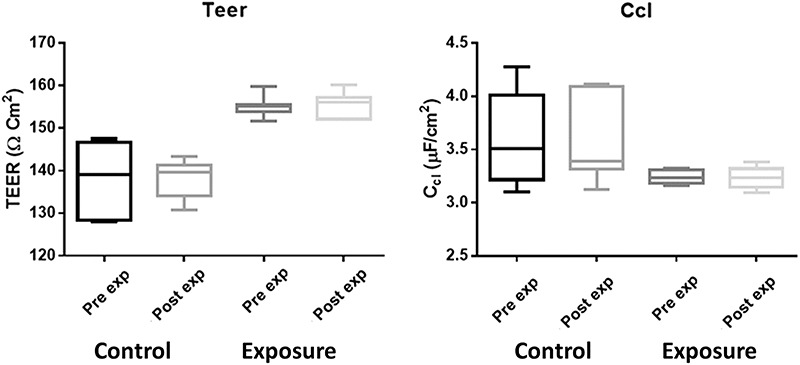
Transepithelial electrical impedance (TEEI) parameters pre- and post-magnetic field treatment of 60 Hz at 10 G.

**Fig. 4.**
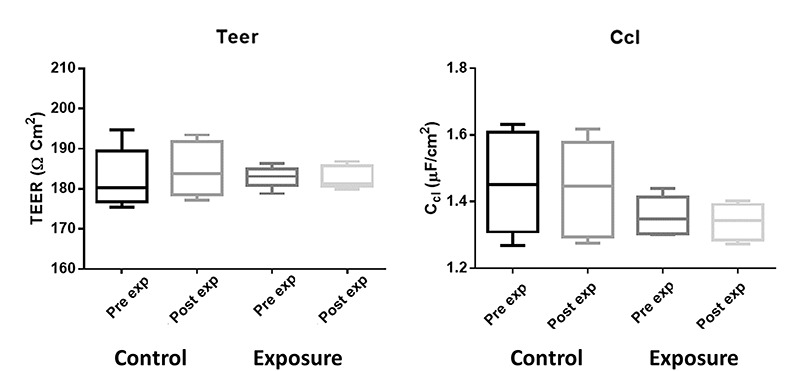
Transephitelial electrical impedance (TEEI) parameters pre- and post-magnetic field treatment of 60 Hz at 50 G .

The confluence percentage was obtained using Equation 1, where A is the area of the wound corresponding to the initial condition *t*_0_, and B is the area after a time, *t*_x_.

(1) \begin{equation*} \%\mathrm{confluence}=\frac{\left(\mathrm{A}-\mathrm{B}\right)}{\mathrm{A}}\times 100 \end{equation*}

### Immunofluorescence

To measure immunofluorescence, cells were grown on round coverslips (~8 mm diameter) and placed on slides for observation under the confocal microscope.

The expression of the CLDN-1 and ZO-1 proteins of the tight junction was determined by immunofluorescence and used to evaluate the effects of magnetic field radiation exposure on epithelia [27]. The results were obtained using a confocal microscope (TSP2, Leica, Germany), by observing antibodies and fluorophore at 520 nm. The immunofluorescence technique also enables the evaluation of changes in the shape and size of the cell nuclei. For this, the DAPI fluorophore (4′,6-diamidino-2-phenylindole; 10 mg ml^–1^ in H_2_O stock solution; Invitrogen D1306) was used.

The maximum intensity projection (MIP) was obtained from a *Z*-stack of eight image slices on the confocal microscope. Pixel histograms of the fluorescent expression of the tight junction proteins were generated from this, using Zen 2.3 lite software (Carl Zeiss, Germany). The shape (circularity), size and density of the nuclei were also determined, using the MATLAB ‘regionprops’ function [25].

MDCK cell groups were used for the control and exposure groups. The experiment was carried out in two sets of six exposures. For CLDN-1, a magnetic field of 60 Hz at 50 G was applied every 4 h for 60 min each, for 24 h exposure. For ZO-1, the cycle was as follows: 24 h exposure; 24 h no exposure; 24 h exposure. Sampling for this experiment was performed at 24, 48 and 72 h.

**Fig. 5.**
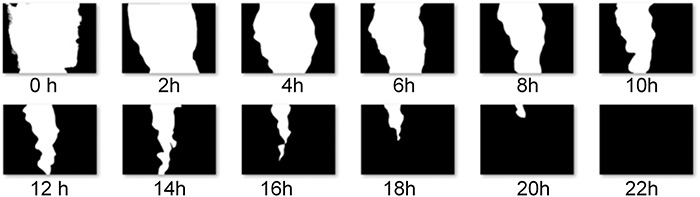
Sequence of the binarized images of the wound healing evolution for cells exposed to the magnetic field.

**Fig. 6.**
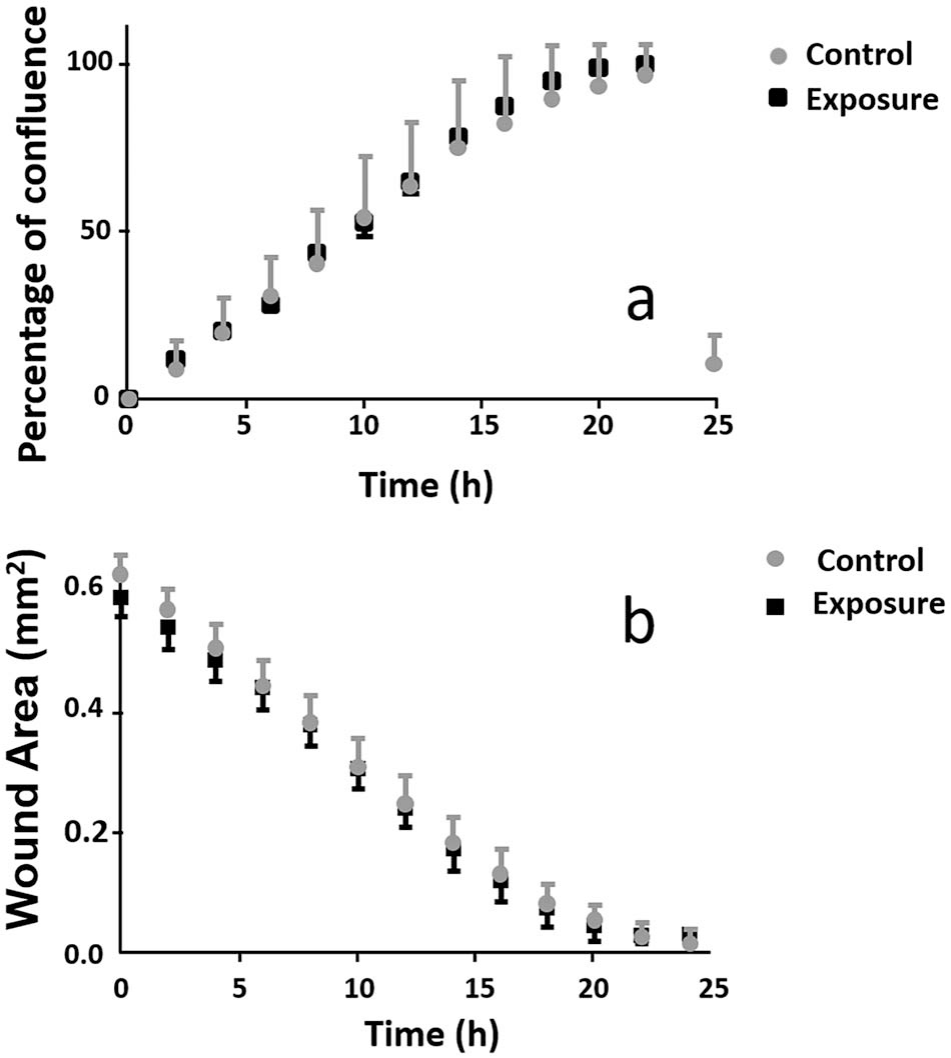
(a) Confluence percentage of the cell monolayers results; (b) the evolution of the area corresponding to the wound closure.

## RESULTS

### TEEI

The TEEI results corresponding to the magnetic field exposure tests of 60 Hz at 10 and 50 G for the cell monolayers are shown in Figs 3 and 4, respectively.

A paired Student’s *t*-test was performed (GraphPad, Prism), so differences before and after exposure to 60 Hz at 10 G could be determined. The TEER and CcL results, in both the control (p_TEER_ = 0.7046 and p_Ccl_ = 0.4856) and the exposed (p_TEER_ = 0.7410 and p_Ccl_ = 0.9096) groups, were not statistically different.

Similarly, the *P* values of TEER and Ccl, before and after the magnetic exposure to 60 Hz at 50 G , were not significantly different for the control cells (pTEER = 0.3943 and pCcl = 0.2213) or the exposed cells (pTEER = 0.6089 and pCcl = 0.0121).

### Scratch-wound assay

This assay was carried out with eight samples.

Figure 5 shows that the sequence of binarized images produced by MATLAB corresponds to the evolution of the wound closure, as a result of the confluence of a cellular monolayer exposed to magnetic fields.

Figure 6a shows the results of the confluence percentage of the control and treated cell monolayers, and Fig. 6b shows the wound area.

**Fig. 7.**
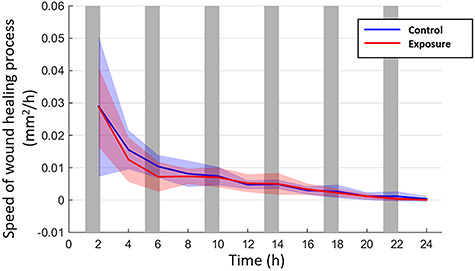
The trend of speed confluence during the cellular migration and proliferation processes.

Figure 7 shows the speed of the confluence of cell monolayers; the evolution of the area corresponding to the wound healing. It also shows the tendency of the confluence rate of the cell monolayer. It should be noted that the wound area and the speed decreased over time.

### Immunofluorescence

**Fig. 8.**
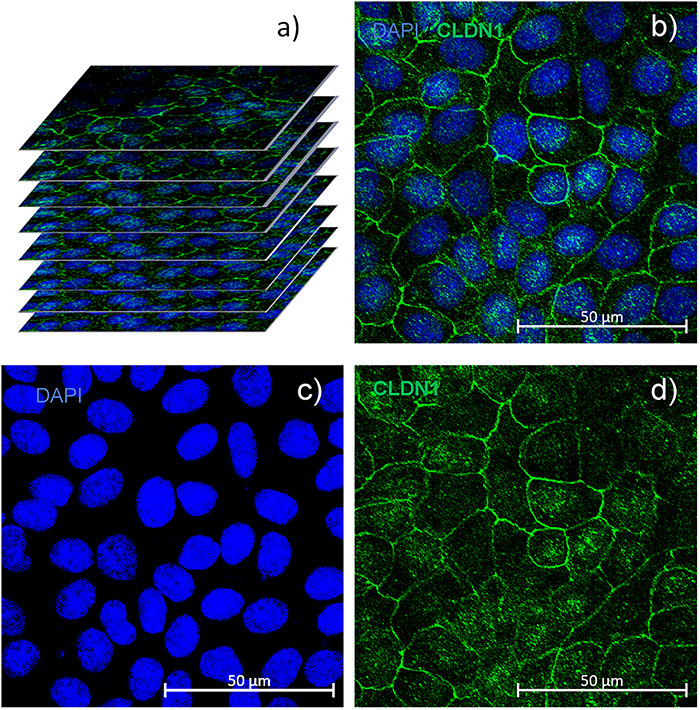
Immunofluorescence assay from (a) a *Z*-stack of eight slices by confocal microscopy; (b) maximum intensity projection; (c) cell nuclei; (d) expression of tight junction proteins CLDN-1 and ZO-1.

Figure 8a shows the *Z*-stack of eight slices of microscope images. Figure 8b shows the MIP, which corresponds to the sum of the pixel intensities of the slices. Figure 8c and d show the cell nucleus and the expression of the CLDN1 protein of the tight junction, respectively.

### Fluorescence intensity of CLDN-1 and ZO-1

**Fig. 9.**
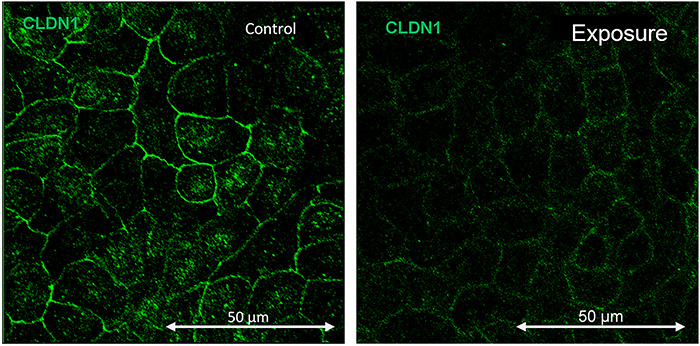
Immunofluorescence for the maximum intensity projection-related CLDN1 from exposure to 60 Hz at 50 G electromagnetic frequency (ELF-MF).

Protein expression is proportional to the fluorescent proteins in the tight junctions. In this study, the parameter was determined by the number of color pixels corresponding to the fluorophore used, which represents the intensity. The fluorescent image of CLDN-1 expression after exposure to 60 Hz ELF-MF at 50 G can be seen in Fig. 9.

**Fig. 10.**
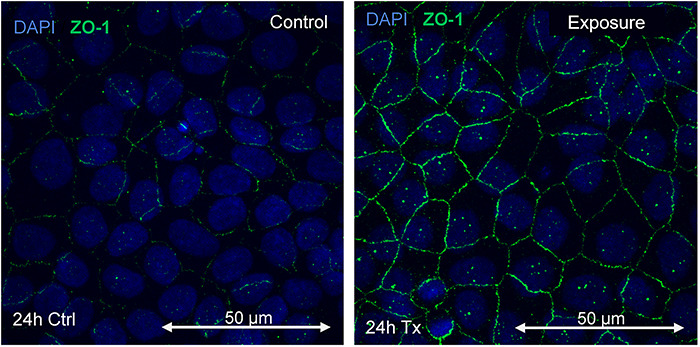
Immunofluorescence of control and treatment in the first 24 h of 60 Hz at 50 G magnetic field exposure.

**Fig. 11.**
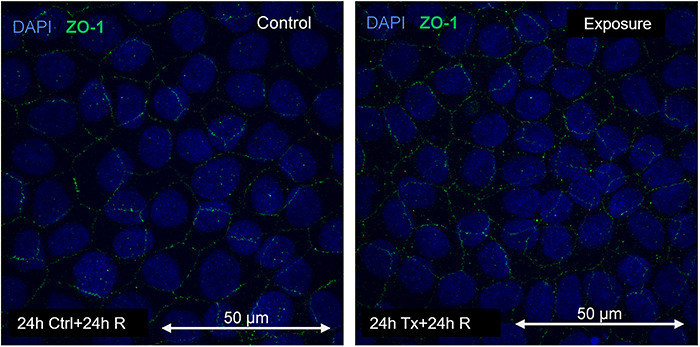
Immunofluorescence of control and treatment at 48 h of magnetic field exposure (24 h of exposure + 24 h of rest) to 60 Hz at 50 G .

**Fig. 12.**
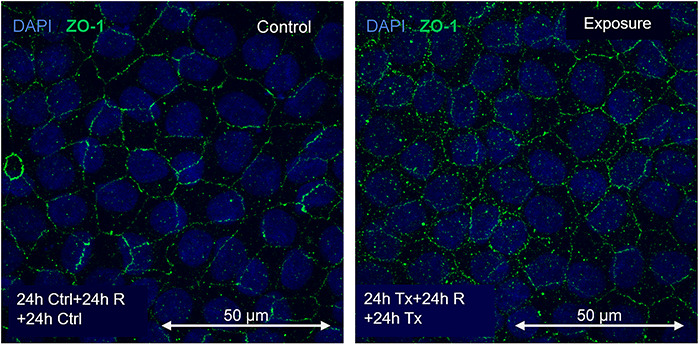
Immunofluorescence of control and treatment at 72 h of magnetic field exposure (24 h exposure + 24 h rest + 24 h treatment) to 60 Hz at 50 G .

The results of the MIP of ZO-1 expression are shown in Figs 10, 11 and 12.

Immunofluorescence of the control and exposure at 48 h of exposure (24 h exposure + 24 h no exposure) to a 60 Hz magnetic field at 50 G is shown in Fig. 11.

Immunofluorescence of the control and exposure at 72 h of exposure (24 h exposure + 24 h no exposure + 24 h exposure) to a magnetic field of 60 Hz at 50 G is presented in Fig. 12.

**Fig. 13.**
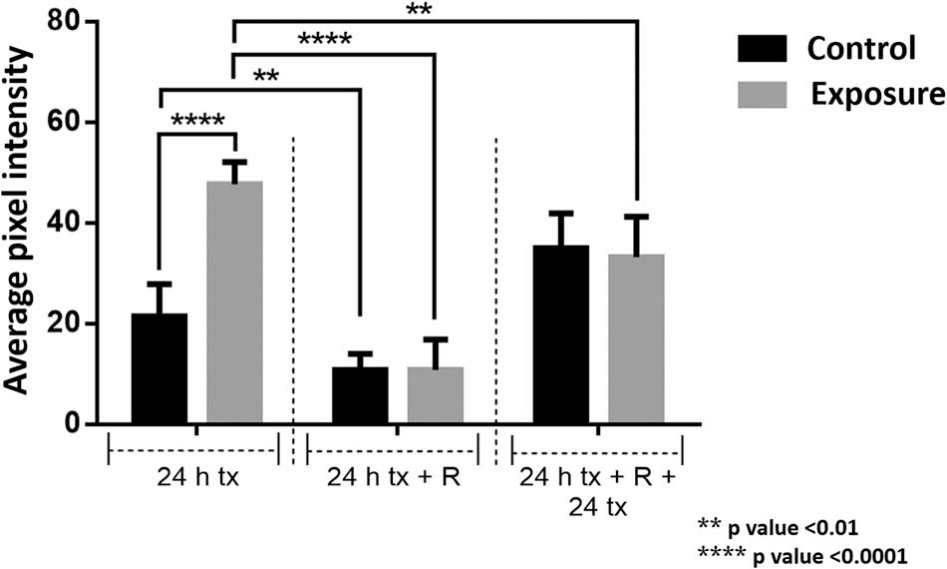
A comparative graph of the average pixel intensity related to the presence of ZO-1 with exposure to 60 Hz at 50 G. D = 24 h without exposure

A comparative graph of the average intensity of pixels related to the presence of ZO-1 after exposure to 60 Hz at 50 G is shown in Fig. 13. The effect of the magnetic field at the beginning of the exposure (first 24 h) is worth noting.

### Cell nuclei characteristics

**Fig. 14.**
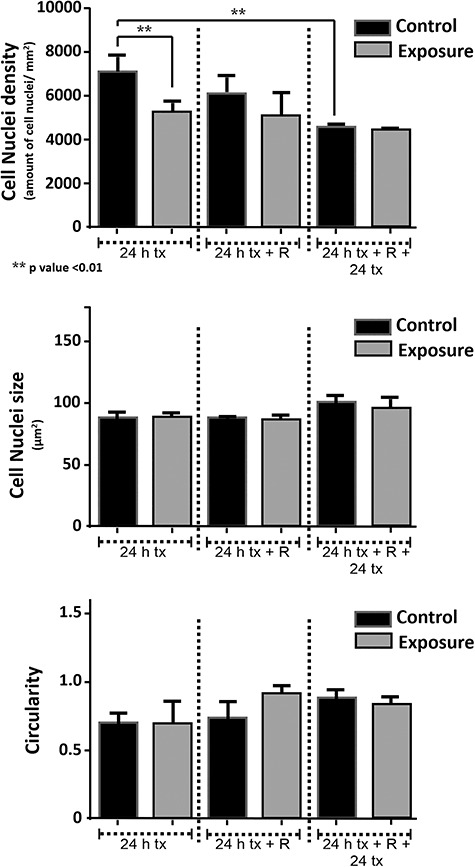
(a) Cell nuclei density over 24 h; (b) cell nuclei area over 24 h; (c) cell nuclei circularity over 24 h.

Figure 14a shows the densities of the cell nuclei of the images obtained in the immunofluorescence experiment.

The area and shape analyses of the cell nuclei are shown in Fig. 14b and c, respectively. The cell density parameter was obtained by automatic cell counting of the cell nuclei, assuming that each cell has a single nucleus. The results correspond to samples taken every 24 h, as previously described. The analysis showed a significant decrease in the exposed population during the first 24 h.

## DISCUSSION

### TEEI

The application of magnetic fields of 60 Hz at 10 and 50 G in confluent cell monolayers of the MDCK cell line did not have a significant effect on TEEI. However, since the expression of the proteins in tight junctions was significantly altered, it can be inferred that TEEI should also be modified with exposure to magnetic fields. Therefore, a re-evaluation of the effect of TEEI should be conducted, considering the exposure conditions used in the determination of protein expressions.

### Wound closure

Cell migration and the confluence of the cell monolayer showed no significant changes with exposure to a magnetic flux density of 50 G at 60 Hz. These results were similar to those reported when a 30 mm^2^ circular wound was made in epithelia of the human respiratory system, without exposure to magnetic fields [25]. If experiments were performed with cell lines with confluence rates lower than MDCK, as suggested in Manzanares *et al*. [23], then magnetic field effects could potentially be observed because the exposure time would extend over 24 h. It would also be possible to follow the evolution of the process in detail.

Regarding the indicator of migration and cell proliferation [28], contact inhibition was observed. This phenomenon is lost in cells with uncontrolled behavior, which could eventually produce benign or malignant tumors in the tissues. Therefore, the conservation of the phenomenon of contact inhibition in the cell cultures exposed to magnetic fields suggests that no adverse effects were induced in the treated cultures. This result is relevant and should be verified in subsequent studies.

### Immunofluorescence

The expression of CLDN-1 decreased by 90% compared with the control, while for ZO-1 it increased by >120%. It should be noted that these changes occurred during the first 24 h of exposure. In this time, cells were exposed to the magnetic field after one-third of the epithelium was removed, fixed and subjected to fluorescence measurement. This population exhibited significant effects on exposure. After 24 h without exposure, the fluorescence decreased, which could suggest a restoration of protein concentration in the tight junctions. The second exposure to magnetic fields, between 48 and 72 h, did not significantly alter the protein levels in the junctions. This could indicate not only a tissue recovery process due to the rest period, but also an adaptation process, demonstrating that exposure to magnetic fields alters the processes that develop in tight junctions, in addition to possible recovery and adaptation reactions.

Cell density was obtained by automatic counting of cell nuclei, assuming that each cell had only a single nucleus. The results showed a significant decline in the population after the first 24 h of exposure. This may be related to the scratch-wound assay, where a higher confluence speed was obtained in the early hours of magnetic field exposure, which then decreased significantly after 6 h of exposure (Fig. 7). The effects on cell density, due to the presence of magnetic fields, were evaluated by comparing the values obtained before and after exposure. So, if the stages of increase and decrease in cell production occur during the exposure and not at the end, where the confluence speed decreases significantly, it could explain the reduction in cell density.

There were no statistically significant differences between the control and exposed cells in terms of area and shape of the nuclei.

## CONCLUSIONS

The parameters proposed as indicators to observe the possible effects of magnetic exposure on epithelial cells were those associated with electrical (TEEI), physiological (cell proliferation and migration, expression of the CLDN-1 and ZO-1 tight junction proteins) and physical (changes in density, shape and size of the nuclei) phenomena.

Concerning TEEI and cell migration proliferation, no significant effects were observed due to magnetic field exposure. The evaluation of the proteins of the tight junction by immunofluorescence found significant differences during the first 24 h of magnetic field exposure. Significant differences were observed in the density of cell nuclei, but not in their shape or size.

These experiments, carried out in controlled environments and under physiological conditions, ensure that the observed effects were strictly due to exposure to magnetic fields. The infrastructure developed for this project will enable continued experimentation with the biological structure used in this study or with others where it has been reported that they are affected by the presence of magnetic fields.
